# Michigan Dental Association

**Published:** 1874-12

**Authors:** Thomas R. Perry


### Proceedings of Societies.

### MICHIGAN DENTAL ASSOCIATION.

The Michigan Dental Association met Oct. 13th, 1874, in Ypsilanti. Meeting called to order at 12 m. by President Hawxhurst. Owing to the absence of some of the officers it was deemed best to adjourn until 2 p. m., when the President again called the meeting to order.

On motion of W. H. Jackson, the regular order of business was suspended until 7 p. m., to conduct clinics, which occupied the afternoon.

### EVENING SESSION, 7 P. M.

The different standing committees not being ready to report were given until next day. The Association then proceeded to ballot for a place for holding the next meeting, which resulted in favor of Grand Rapids. The time, second Tuesday in October, 1875. The election of officers for the ensuing year resulted as follows:

For President, Thomas R. Perry, Grand Rapids; Vice-President, J. W. Finch, Adrian; Secretary, W. D. Tremper, Ypsilanti; Treasurer, E. S. Holmes, Grand Rapids; Censor, J. A. Watling, Ypsilanti. The board of censors is as follows: J. A. Watling three years, G. R. Thomas two years. D. C. Hawxhurst one year.

E. A. Post, M. D., of Ypsilanti, was introduced to the Association and made some very interesting remarks on the subject of dental education, advocating a law that would protect the people from quackery, not only in the dental but medical profession. He spoke in very complimentary terms of the progress that dentistry had made in the last few years; he could remember when dentistry was entrusted to barbers and village blacksmiths, thanks to the educated, that day has past. To still further advance the standard of the profession was to educate the people. The Secretary here read an invitation from Dr. and Mrs. Watling to meet them at their residence at 8 o'clock, Wednesday eve.

Dr. Holmes, Chairman of Committee on University to establish a dental department, reported progress. The regents and professors are in favor of the movement, but have not the funds for that purpose. He proposes that a petition be presented to the legislature to collect a special tax for that purpose. The committee concludes that they can not do any thing further until the session of legislature, when they should labor with that body.

W. H. Jackson spoke on the same subject and said, the regents for the want of proper funds would not start a new department.

Dr. Watling thinks the regents do not act in good faith, for they found funds to introduce ladies into the University.

Dr. G. L. Field is opposed to the movement, thinks we have too many dental colleges to support, and should not establish any more until we can maintain those already established. Thinks a thorough dental education can be obtained at an established college.

W. H. Jackson thinks we ought to have a place where a thorough dental education can be obtained before we pass a law to prevent qnacks from practicing in our state.

Dr. Watling thinks a more thorough preparation to practice dentistry can be obtained at the University, with proper teachers, than can be at a dental college.

Dr. Douglass coincides with Dr. Watling. He said a year ago he consulted Prof. Taft about a student first attending university, then college; he replied that it was a good plan to get a good education.

Dr. Post said a dental department would be of benefit, but how can it be gained? Thinks representatives a poor place to carry a petition to unless signed by the people, which is a loud cry and would be heeded. He advised continual application for recognition as a profession, and it must meet with final success.

Dr. Field said the legislature had been applied to to admit the Homœopathists by strong petitions from the people, and bill passed but the regents refused to act.

Dr. Thomas fully agrees with Dr. Holmes, if we are a branch of medicine he sees no reason why we ought not to be sustained in our efforts in gaining a good education. If the dental colleges do not have better teachers they ought not to succeed. Thinks a department in University will stimulate the dental colleges, and make them better; advocates the raising of a fund, by the dentists, to establish a department in the University; hopes the report of committee will be adopted. Adjourned to 9 a. m.

### MORNING SESSION

President in chair, minutes read and approved. The report of Committee on University was accepted and committee continued.

Dr. Watling, Chairman of Committee on Dental Education, made a report which brought out considerable discussion.

W. H. Jackson: We should all understand and have a full knowledge of medicine before we can practice successfully in our specialty, and until we gain a high standard of excellence we can not hope to be recognized by medical men as a profession. Dr. Field: College education does not make a dentist always, knows of cases where college graduates are a disgrace to the profession. Dr. Holmes is glad to have opposition for it brings out facts, hopes to hear from both sides, is in favor of gaining all the light from college influence; wants our own University to be the leading institution in the country for gaining a thorough dental education, it will have a tendency to drive quackery from our midst. Dentistry is not a trade, but must be looked up to as a scientific calling, and hopes we will all take an interest in dental education. Dr. Field does not wish to be understood that he opposes a thorough dental education. He wishes we had a better class of men in our ranks. The reason the colleges do not send out better dentists, is because we do not send good enough students; more colleges will lessen the standard of education. Dr. Holmes thinks a department at the University will make better dentists, and hoped for success in gaining such a department. Dr. Thomas thinks more schools will not have a tendency to lower the standard of education, but on the contrary elevate it to a higher standard; has not so much faith in laws enacted to prevent quacks, but thinks the only way to be a thorough dentist is to be thorough in our education. Dr. Watling thinks that the strife between the colleges too great to make good practical dentists. Dr. Jackson thinks we ought to understand medicine enough to treat all abnormal conditions of the mouth suecessfully. Dr. Holmes: Dentistry is not a specialty of medicine any more than medicine is of dentistry; does not like the term specialty of medicine, we are all working to the same end, curing our fellow men. The treatment of the teeth is surgery as much as is the treatment of any other organs by the general surgeon. Dr. I Douglass does not like the term specialists. We, as surgeons, perform operations which the general surgeon does not and can not; thinks we we ought to gain a better knowledge of medicine.

Dr. Hawxhurst read an extract from REGISTER, wherein the article advocates a thorough medical education to successfully follow our vocation. In his remarks he cited cases where dental students, in Europe, passed better examinations than medical students. The standard of dental education is being elevated, and believes in exerting ourselves to gain a higher place in the sciences. Dr. Douglass thinks the surest way to gain a better knowledge of dental education, is to establish a dental department in the University.

Dr. E. S. Holmes, Chairman of Committee on Anatomy stated that he had been prevented, by ill health, from preparing a report worthy of the name, but presented a hastily prepared paper on the subject. He said every dentist should be intimately acquainted with anatomy for the more successful treatment of abnormal conditions of the teeth, also should have a full anatomical knowledge of nerves and muscles.

Hopes all tutors will see that their students are familiar wtih anatomy for unless they are, they must necessarily be superficial. After a few remarks by Drs. Watling, Douglass and Jackson the subject was passed.

### PHYSIOLOGY.

W. H. Jackson, of Committee on Physiology, read a very interesting paper on the subject, with explanatory diagrams. E. S. Holmes, Chairman of Committee on Publishing Manual, made a verbal report which was accepted. At his own request he was discharged. He was requested to name his successor. Adjourned to 2 p. m.

### AFTERNOON SESSION.

Meeting called to order by President, minutes read and approved. The following delegates were elected to represent this body at the American Dental Association of 1875: W. D. Tremper, J. H. Woolley, W. L. Andrews, W. P. Morgan, J. W. Finch, T. R. Berry. On motion of Dr. Thomas, the motion to relieve Dr. Holmes from Chairman of Committee on Manual be recinded. Carried. Dr Tremper, Chairman of Committee on Pathology, read a very able paper on the pathological conditions of the mucous membrane. After a short discussion the subject passed. Dr. I. Douglass presented a tooth which he had extracted, and found the root had been so disintegrated that the crown separated at the neck. The patient had been taking potash as a medicine for some time, could not say whether that had anything to do with it or not, but it was worthy of consideration.

Dr. Douglas, of Committee on Surgery, read quiet a lengthy paper on that subject, giving many cases and their treatment. A short discussion followed when the subject was passed. On motion of Dr. Watling, the delegates to the American Dental Association were instructed to vote for the amendment to the constitution, making graduates only eligible to membership. Adopted. The Association then listened to the retiring President's address. A letter was received from Dr. Keely in reference to Dr. Barnum. Dr. Robinson offered the following which was adopted: *Resolved* that every member of this Association contribute all he can to the Barnum Fund. On motion the meeting adjourned.

THOMAS R. PERRY, Sec.

